# Changes in the Relative Balance of Approach and Avoidance Inclinations to Use Alcohol Following Cue Exposure Vary in Low and High Risk Drinkers

**DOI:** 10.3389/fpsyg.2017.00645

**Published:** 2017-05-08

**Authors:** Ross C. Hollett, Werner G. K. Stritzke, Phoebe Edgeworth, Michael Weinborn

**Affiliations:** ^1^Cognition Research Group, School of Arts and Humanities, Edith Cowan University, JoondalupWA, Australia; ^2^School of Psychological Science, University of Western Australia, CrawleyWA, Australia

**Keywords:** alcohol, ambivalence, approach, avoidance, craving, cue-reactivity, *ad libitum*

## Abstract

According to the ambivalence model of craving, alcohol craving involves the dynamic interplay of separate approach and avoidance inclinations. Cue-elicited increases in approach inclinations are posited to be more likely to result in alcohol consumption and risky drinking behaviors only if unimpeded by restraint inclinations. Current study aims were (1) to test if changes in the net balance between approach and avoidance inclinations following alcohol cue exposure differentiate between low and high risk drinkers, and (2) if this balance is associated with alcohol consumption on a subsequent taste test. In two experiments (*N* = 60; *N* = 79), low and high risk social drinkers were exposed to alcohol cues, and pre- and post- approach and avoidance inclinations measured. An *ad libitum* alcohol consumption paradigm and a non-alcohol exposure condition were also included in Study 2. Cue-elicited craving was characterized by a predominant approach inclination only in the high risk drinkers. Conversely, approach inclinations were adaptively balanced by equally strong avoidance inclinations when cue-elicited craving was induced in low risk drinkers. For these low risk drinkers with the balanced craving profile, neither approach or avoidance inclinations predicted subsequent alcohol consumption levels during the taste test. Conversely, for high risk drinkers, where the approach inclination predominated, each inclination synergistically predicted subsequent drinking levels during the taste test. In conclusion, results support the importance of assessing both approach and avoidance inclinations, and their relative balance following alcohol cue exposure. Specifically, this more comprehensive assessment reveals changes in craving profiles that are not apparent from examining changes in approach inclinations alone, and it is this shift in the net balance that distinguishes high from low risk drinkers.

## Introduction

Alcohol craving is now recognized as a diagnostic feature in substance use disorders, (DSM-5; American Psychiatric Association, 2013) and is associated with a wide range of treatment outcomes, such as engagement (Schlauch et al., 2012), relapse (Miller et al., 1996; Law et al., 2016) and drinking behavior (Klein et al., 2007; Klein and Anker, 2013; Schlauch et al., 2015b; McHugh et al., 2016). Accurate assessment of craving is important, because reductions in craving can explain up to 50% of treatment effectiveness (Subbaraman et al., 2013).

While craving has been traditionally captured by rating the desire to use alcohol, i.e., from “none” to “intense” (e.g., Payne et al., 1992; Coffey et al., 1999), there has been a shift toward using multidimensional scales to assess both approach (strength of desire to consume) and avoidance (strength of desire to resist consumption) inclinations (Connolly et al., 2009; Jones et al., 2013; Di Lemma et al., 2015; Schlauch et al., 2015b). The ambivalence model of alcohol craving (Breiner et al., 1999) offers a framework for conceptualizing these two dimensions and, importantly, for disambiguating ambivalent (high levels of both approach and avoidance inclinations) and determined (e.g., high approach coupled with low avoidance) motivational craving states. That is, different craving profiles might be observed when individuals are exposed to alcohol cues, not only because one or both inclinations may change following cue exposure, but those changes may also result in a shift in the relative strength of approach versus avoidance over time. If, after cue-elicited changes in craving, the strength of an approach inclination outweighs the strength of an avoidance inclination, the risk of unrestrained and potentially harmful drinking episodes increases. In contrast, if approach inclinations substantively increase after cue exposure, but the increase in cue-elicited approach craving is balanced by an equally strong avoidance inclination, such ambivalent craving may prompt restraint and resistance to drinking. The aim of the present studies was to examine if cue-elicited changes in approach and avoidance inclinations result in different craving profiles for low and high risk drinkers, and subsequent drinking behavior in a lab-based alcohol taste test.

The ambivalence model of alcohol craving (Breiner et al., 1999) proposes that craving consists of separate ***approach*** and ***avoidance*** dimensions. Therefore approach and avoidance exist simultaneously and, depending on the relative strength of each dimension, may compete, or even balance one another. That is, if the avoidance inclination is low, the approach inclination is unimpeded, and even moderate approach inclinations may increase likelihood of alcohol use. Conversely, if the avoidance inclination is high, it may counteract the approach inclination even if it is also high and hence may decrease the likelihood of alcohol use. Therefore if only the approach inclination is measured, individuals with these profiles would appear the same. Such an assessment would fail to detect and quantify the ambivalence experienced by individuals with the second profile. This would be a critical factor in terms of predicting subsequent drinking behavior and treatment readiness and engagement (Miller and Tonigan, 1996; DiClemente et al., 2004; Schlauch et al., 2012). Recent latent profile analyses of reactivity to alcohol cues (Schlauch et al., 2015b) showed that patients in an acute detoxification facility exhibited distinct motivational craving profiles reflecting differing individual patterns based on approach and avoidance inclination levels: (1) approach (high approach, low avoidance), (2) avoidance (low approach, high avoidance), (3) indifference (low approach, low avoidance), and (4) ambivalence (high approach, high avoidance), with ambivalence further split into moderate and intense sub-profiles. Importantly, patients with the three craving profiles where approach inclinations were countered by equally strong or higher avoidance inclinations were more likely to have sought voluntary admittance to treatment (Schlauch et al., 2012, 2015b).

The validity of the ambivalence model has been supported across both clinical and non-clinical samples. For instance, in patients being treated for alcohol dependence, Klein et al. (2007) found that higher approach inclinations were related to dependence severity, and higher avoidance was related to greater time since last drink and fewer drinks in the past week. The positive relationship between avoidance and time since last drink was replicated by Klein and Anker (2013) in an outpatient sample. In contrast, approach inclinations were negatively associated with abstinence and positively associate with drinks per day (Schlauch et al., 2012; Klein and Anker, 2013). That is, if left unrestrained, approach inclinations appear to facilitate alcohol use, thus reducing the likelihood of successful treatment outcomes. Further, avoidance is also positively correlated with number of sessions attended (Schlauch et al., 2012), suggesting that it enhances treatment engagement.

Indeed, a growing literature supports that craving profiles high in avoidance are distinct and lead to fewer lapses and further advancement in recovery. In a sample of alcohol-dependent patients in treatment (Stritzke et al., 2007), who were categorized into three subgroups of high lapsers (used alcohol daily or most days of the week), low lapsers (used alcohol between 2 and 3 times a month), or abstainers (used no alcohol during treatment), low lapsers typically exhibited a profile of ambivalence – that is, scoring highly on both approach and avoidance. In contrast, high lapsers exhibited a profile of dominant approach compared to avoidance, whereas abstainers exhibited a profile consistent with dominant avoidance compared to approach. A longitudinal study has yielded comparable conclusions when exploring the relative impact of avoidance on approach inclinations over the course of 6 months in a large patient sample diagnosed with alcohol dependence and mental illness (Schlauch et al., 2013). Avoidance moderated approach inclinations such that the relationship between approach and subsequent drinking was attenuated. Moreover, high avoidance and low approach were associated with decreased drinking over time, and decreases in drinking predicted higher subsequent avoidance inclinations.

However, other research approaches are needed to extend these findings. Experimental exposure to alcohol cues offers the opportunity to examine the relative strength of approach versus avoidance in real time. In a recent study by Di Lemma et al. (2015), approach and avoidance inclinations were captured in heavy drinkers before and after exposure to positively- or negatively-valenced alcohol videos. Across three separate samples, the craving scores confirmed that the positive alcohol video elicited increases in approach and decreases in avoidance with the converse pattern observed for a negative alcohol video, supporting the independence of the craving dimensions. However, results also supported that, compared with approach and avoidance scores *before* the cue exposure, there was also an exposure-related change in *the pattern of the relative strengths* of approach and avoidance inclinations. That is, across all three samples, prior to the negative video exposure, approach inclinations were considerably higher than avoidance inclinations. By contrast, the craving profile following exposure was characterized by *equivalent* approach and avoidance inclinations, suggesting a shift toward a balanced craving profile. This distinct and consistent shift in relative balance could be as critical for understanding craving when exposed to alcohol cues as the change in the individual magnitude of approach or avoidance. Moreover, variability in the net balance between approach and avoidance inclinations may influence whether alcohol consumption occurs at high or low levels of risk.

## Study 1

The aim of Study 1 was to test if approach and avoidance inclinations before and after alcohol cue exposure differ between high and low risk drinkers. The Australian Government National Health and Medical Research Council [NHMRC] (2009) guidelines defining risky drinking as exceeding 14 standard drinks per week were used to recruit high and low risk drinkers. A standard drink in Australia corresponds to 10 g of alcohol (National Health and Medical Research Council [NHMRC], 2009). To capture approach and avoidance inclinations before and after cue exposure, two single item scales (one for approach and one for avoidance) were administered (Stritzke et al., 2004; Connolly et al., 2009). This allowed for monitoring of relative changes throughout alcohol cue exposure so that the *net* craving magnitude (that is, balanced versus other craving profiles) could be estimated, and differences between high and low risk drinkers in the transient nature of approach and avoidance inclinations across an alcohol cue reactivity period could be captured. From this perspective, craving is best quantified by considering the balance of the restraining influence of the avoidance inclination against the strength of the approach inclination.

In line with the ambivalence model, drinkers who differed in the degree of risky alcohol use were anticipated to demonstrate different patterns of approach and avoidance inclinations before and after exposure to alcohol cues. According to unidimensional (approach only) accounts of craving, assessment of avoidance is irrelevant (Kavanagh et al., 2013). In contrast, the ambivalence model of craving considers avoidant inclinations, and the only cue reactivity pattern where avoidance strength does *not* add important information occurs when high risk drinkers compared to low risk drinkers show both a greater increase in approach and a greater decrease in avoidance, assuming similar levels of approach and avoidance at baseline. Therefore a unidimensional account of craving would predict that:

(1) Assessment of changes in approach strength alone would be sufficient to distinguish between craving profiles in high and low risk drinkers.

However, there are at least two alternative cue reactivity patterns where the failure to assess changes in avoidance strength would result in an incomplete understanding of the manner in which high risk drinkers differ from low risk drinkers in their craving strength. Therefore, by assessing avoidance inclinations before and after cue exposure, two additional potential patterns of interest would be:

(2) Both high and low risk drinkers would show a similar increase in approach inclinations following cue exposure, but what distinguishes their reactivity profiles is that only high risk drinkers would show a simultaneous decrease in avoidance inclinations. That is, there would be a larger net increase in the weight of approach relative to avoidance in the high risk drinkers compared to low risk drinkers.
(3) If the relative strength of approach and avoidance differs between the groups at baseline, similar changes in each dimension can result in a different *relative balance* of approach and avoidance strength across both groups *after* cue exposure. For example, if both groups show higher avoidance than approach inclinations at baseline, but this difference is much smaller in the high risk drinkers, then even with similar changes in each dimension following exposure there would be a larger net increase in approach relative to avoidance for the high risk drinkers. That is, for the high risk drinkers an increase in approach could outweigh avoidance after exposure, whereas for the low risk drinkers (due to the initial larger dominance of avoidance over approach) the same increase in approach could still be balanced by equally strong avoidance following cue exposure.

Based on the ambivalence model of craving we hypothesized that, compared to low risk drinkers, for high risk drinkers alcohol cue exposure results in a greater net increase in the strength of approach inclinations relative to the strength of avoidance inclinations.

### Materials and Methods

#### Participants

Participants were 60 (52% female) undergraduate students aged 18–59 (*M* = 21.28, *SD* = 6.93) from the University of Western Australia (UWA) who were 18 years or older, and self-reported consuming at least eight standard drinks per week. The average standard drinks consumed per week (assessed via self-report of quantity/frequency at screening of a pool of students volunteering for research participation credits in psychology units; *N* = 824) were 20.09 (*SD* = 15.23). Participants were split into high (15 males, 12 females) and low (14 males, 19 females) risk drinkers based on the Australian NHMRC guidelines of no more than two standard drinks per day on average for both genders (or 14 drinks per week). This is consistent with recent studies using similar guidelines to establish excessive drinking criteria (Connolly et al., 2013; Jones et al., 2013; Di Lemma et al., 2015). Thirty-three reported drinking 14 or less standard drinks per week (*M* = 9.84, *SD* = 4.27) and were considered low risk, and 27 reported drinking more than 14 standard drinks per week (*M* = 32.50, *SD* = 14.06) and were considered high risk. These groups were equivalent on gender ratio (χ^2^ = 1.03, *p* = 0.311) and age, *M*_Low_ = 21.03 (*SD* = 7.78), *M*_High_ = 21.59 (*SD* = 4.27), *t* = -0.31, *p* = 0.758. Participants were excluded if they had been diagnosed or treated for substance abuse or dependence.

#### Materials

##### Demographic Questionnaire

Several items captured demographic information (age, gender, etc.) and alcoholic beverage preference (beer, premix, or wine).

##### Drinking Behavior Questionnaire (DBQ; McEvoy et al., 2004, Adapted from Calahan et al., 1969).

Two items from the DBQ were used as a screening tool to assess average frequency and quantity of standard drinks consumed during the last week. Quantity and frequency measures have shown good correspondence with more robust timeline follow back methods for estimating alcohol consumption (Sobell et al., 2003), and are thus suitable as brief screening measures. Only participants who had consumed alcohol in the last 3 months were asked to complete the DBQ.

##### Craving assessment

Alcohol approach and avoidance inclinations were measured separately (Stritzke et al., 2004), by giving the following instructions: “Often people who drink alcohol have two voices in their head, one that says ‘I really want a drink right now,’ and another that says ‘I would rather not have a drink right now,’ Please rate how strong each one of those two voices is for you personally right now on a scale from 0 (very weak) to 8 (very strong).” Higher scores for the approach item indicated greater approach inclinations, and higher scores for the avoidance item indicated greater avoidance inclinations.

##### Alcohol craving induction

Three classes of alcoholic beverages (beer, premixed, and wine), each with multiple exemplars were available as craving stimuli; these were Smirnoff vodka, Jim Beam bourbon, and Bundaberg rum as premixed options; white and red for wine; and Corona and Pure Blonde for beer. These beverages were selected based on popular varieties or brands, ensuring there was one popular imported and domestic option (e.g., Corona and Pure Blonde respectively). The main component of the craving induction was a short video (a separate one for each beverage class, depending on preference), which was matched for length, setting, and camera angle. Each video was 5 min in length and consisted of still images portraying people drinking in three different settings (outdoors, at home, and in a public bar) with four images per setting (6 s per image). This was followed by a film portraying the opening, pouring, and drinking of the same beverages in the same three settings. The videos were presented using VLC Media Player.

The layout of the room used for the craving manipulation was arranged to create an atmosphere that would maintain attention on alcohol and give the impression that the participant would be consuming their preferred beverage. An A3 poster next to the computer presented photographic images of the preferred beverage having been prepared for consumption (poured in a glass on a picnic table) as well as images of liquor store shelves (i.e., depicting a variety of brands in one image). Also in view was a bar-sized refrigerator. Cartons of the preferred alcoholic beverage choices were stacked next to the fridge to give the impression that large quantities of alcoholic beverages were obtained for the study and stocked in the fridge. All beverages (except red wine) and appropriate drinking glasses were cooled in the fridge, creating layer of condensation intended to increase appeal.

#### Procedure

##### Screening/recruitment

Participants completed the DBQ screening measure and a basic demographics questionnaire during tutorial classes. Participants meeting the eligibility criteria were contacted by phone or email and scheduled for participation in a timeframe acceptable for alcohol consumption (12 pm onward).

##### Laboratory session

Consenting participants attended the 45-min laboratory session where they initially completed the demographic questionnaire and completed the two-item approach and avoidance craving measure for the first time (Time 1). They were subsequently taken to the induction room (prepared with craving induction materials as described above), where they were seated and advised they would watch a short video with headphones on. Once the video was completed, participants were told that they may or may not be asked to participate in a taste test at the end of the session and then completed the second two-item approach and avoidance craving measure (Time 2). No participants were later asked to participate in an actual taste test, however, the purpose of this implied taste test was to induce the anticipation that alcohol was available for potential consumption (thus maintaining the craving state) throughout subsequent computerized decision making tasks for a different study which lasted between 10 and 20 min. Halfway through the computer session, the researcher retrieved the participant’s preferred alcoholic beverage and glass and positioned them on a serving tray in front of the participant just to the left of the computer monitor. Following the computer task, the participants completed the third two-item approach and avoidance craving measure (Time 3), after which the participant was informed they would not be required to take the taste test but that they would be remunerated with an additional $5AUD for good performance during the computer task (in conjunction with course credit). Finally, the participant was debriefed. These procedures were approved by the UWA Human Research Ethics Committee.

### Results

#### Craving Manipulation and Change in Approach and Avoidance Inclinations

**FIGURE 1:**
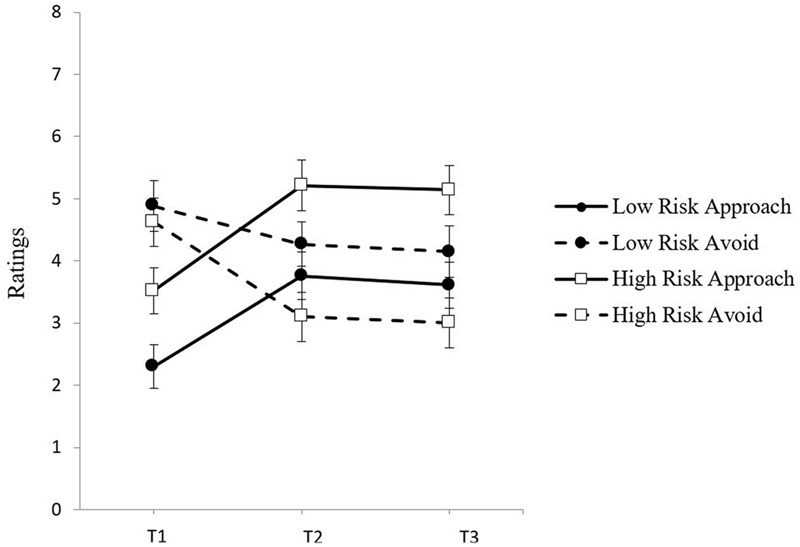
**Mean ratings of approach and avoidance inclinations by group (Low Risk, High Risk) at baseline (Time 1), post-exposure (Time 2), and follow-up (Time 3)**.

To evaluate the pattern of change in approach and avoidance inclinations from pre- to post- craving induction, a 2 (Group: High and Low Risk) × 2 (Time: 1 and 2) × 2 (Inclination: Approach and Avoidance) mixed model ANOVA was conducted with group as the between-groups factor, and time and craving inclination as the within-groups factors. There was a significant Time × Inclination interaction, *F*(1,58) = 53.32, *p* < 0.001, $\eta_{p}^{2}$ = 0.48, indicating that the pattern of change in approach and avoidance inclinations differed following the craving induction. **Figure 1** illustrates that both risk groups showed a pattern of increased approach and decreased avoidance from Time 1 to Time 2. The decrease in avoidance was more than twice as large for the high risk group, *t*(26) = 4.77, *p* < 0.001, *d* = 0.73, than for the low risk group, *t*(32) = 1.74, *p* = 0.091, *d* = 0.28, although the Group × Time × Inclination interaction did not reach significance, *F*(1,58) = 2.84, *p* = 0.097, $\eta_{p}^{2}$ = 0.05. Conversely, the increase in approach was similar for the low risk group, *t*(32) = -5.09, *p* < 0.05, *d* = -0.61, and the high-risk group, *t*(26) = -6.58, *p* < 0.001, *d* = -0.81.

#### Relative Balance Assessment of Approach and Avoidance

To test the prediction of pattern (3) that the relative strength of approach and avoidance inclinations would change from baseline to post-cue exposure, inclinations were examined separately for Time 1 and Time 2, using 2 × 2 (Group × Inclination) ANOVAs. At Time 1, there was a main effect of inclination, *F*(1,58) = 13.34, *p* < 0.01, $\eta_{p}^{2}$ = 0.19, showing that avoidance inclinations were stronger than approach inclinations for both groups. Although the Group × Inclination interaction was not significant, *F*(1,58) = 2.42, *p* = 0.125, $\eta_{p}^{2}$ = 0.04, paired-sample *t*-tests revealed that the low risk group showed greater avoidance than approach at Time 1, *t*(32) = -3.88, *p* < 0.001, *d* = -1.18, whereas for the high risk group this difference between inclinations at Time 1 was smaller and not significant, *t*(26) = -1.41, *p* = 0.169, *d* = -0.49. Examination of effect sizes shows that the difference between inclinations at Time 1 was more than twice as large in the low risk group as compared to the high risk group. At Time 2, the pattern of the relative strength of approach and avoidance inclinations had *reversed* for the two groups as evidenced by a significant Group × Inclination interaction, *F*(1,58) = 7.50, *p* = 0.008, $\eta_{p}^{2}$ = 0.115. Paired samples *t*-tests confirmed that whereas for the high risk group approach was now much stronger than avoidance at Time 2, *t*(26) = 2.96, *p* < 0.01, *d* = 1.04, for the low risk group, approach was still balanced by equally strong avoidance at Time 2, *t*(32) = -0.77, *p* = 0.448, *d* = -0.02.

**FIGURE 2:**
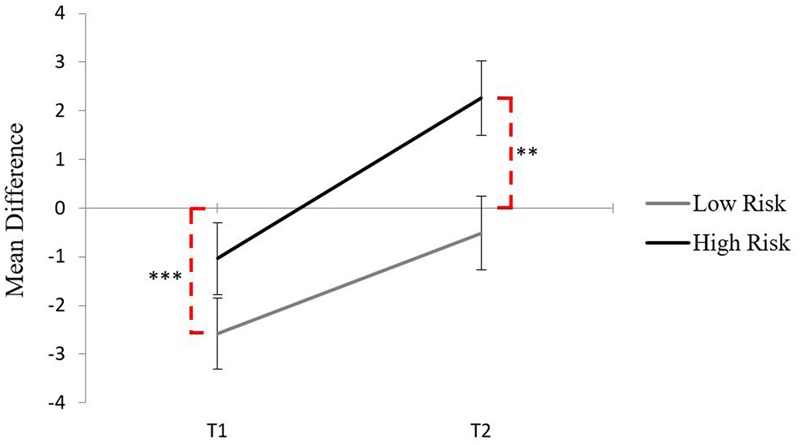
**Mean difference scores for each group across Time 1 and Time 2, dashed lines indicate significance from zero (^∗∗^*p* < 0.01, ^∗∗∗^*p* < 0.001)**.

The change in relative weight can be illustrated by computing approach – avoidance difference scores for Time 1 and Time 2 These difference scores can then be compared to zero to determine if there was any significant deviation in approach or avoidance from a perfectly balanced craving profile. **Figure 2** shows that, at Time 1, for low risk drinkers the negative difference score was significantly different from zero, *t*(32) = -3.88, *p* < 0.001, *d* = -0.67, showing that the balance between approach and avoidance is tipped in favor of avoidance, whereas for high risk drinkers the negative difference score was not significantly different from zero, *t*(26) = -1.42, *p* = 0.169, *d* = -0.27, showing that there was an equal balance between approach and avoidance. At Time 2, while the net strength of approach had increased for both groups, only for the high risk drinkers was there now a positive difference score that was significantly different from zero, *t*(26) = 2.96, *p* < 0.01, *d* = 0.57, showing that the initial balance at Time 1 between approach and avoidance has tipped in favor of approach. In contrast, for the low risk drinkers there was still a negative difference score at Time 2, but it was not significantly different from zero anymore, *t*(32) = -0.77, *p* = 0.448, *d* = -0.13. That is, while the net strength of approach had also increased, it was still balanced by an equally strong avoidance inclination.

### Discussion

According to a unidimensional conceptualization of craving, or pattern (1), high risk drinkers would show both a greater increase in approach and a greater decrease in avoidance than low risk drinkers, assuming similar levels of approach and avoidance at baseline. In this scenario, assessment of changes in approach strength alone would be sufficient to distinguish between motivational craving states in high and low risk drinkers. However, the pattern in the current results does not support this scenario. These findings instead are more in line with pattern (2), because high risk drinkers significantly decreased in avoidance, whereas low risk drinkers only diminished marginally in avoidance from baseline levels. That is, both groups showed similar increases in approach from baseline to post-cue exposure, but could be distinguished by a differential change in avoidance. However, in addition, the risk groups differed at baseline. While both groups initially had higher avoidance than approach scores, this difference was much smaller for the high risk group. This is consistent with pattern (3) where it is the shift in *relative balance* of approach and avoidance that distinguishes between risk groups.

Thus the change in craving state for the high risk drinkers was characterized by a shift from ambivalence to predominantly approach, whereas for the low risk drinkers the shift was one from predominantly avoidance to ambivalence. In sum, the groups could not be distinguished in terms of changes in the approach dimension of craving alone, but differences emerged only when taking into account simultaneous changes in the relative strength of the avoidance dimension of craving before and after cue exposure.

Following the craving induction in Study 1, the craving profile of high risk drinkers was akin to ‘full throttle’ whereas the craving profile for the low risk drinkers was tempered with one foot on the brake. These patterns are consistent with recent findings that alcohol dependent individuals showed weaker implicit negative alcohol associations than social drinking controls, despite sharing similar implicit positive alcohol associations during an implicit association test (Dickson et al., 2013). This imbalance in automatic alcohol cognitions in alcohol dependent individuals compared to the more balanced profile of controls aligns closely with the explicit patterns of approach and avoidance observed in high and low risk drinkers in the present study following cue exposure. Dickson et al. (2013) similarly interpreted the stronger negative alcohol associations observed in social drinking controls in their study as a ‘protective brake.’ Based on these findings, activation of cue-elicited avoidance inclinations would require more effort in high risk drinkers than in low risk drinkers. That is, when faced with alcohol cues that elicit an increase in approach inclinations, it is easier for low risk drinkers than for high risk drinkers to maintain the balancing counterweight of an avoidance inclination. This might explain why low risk drinkers maintained higher avoidance than approach throughout the craving induction in the present study, whereas high risk drinkers failed to do so.

Study 1 had several limitations. The validity and utility of a theoretical conceptualization of craving lies in its ability to predict drinking (Kavanagh et al., 2013). Therefore, in Study 2 an *ad libitum* taste test as a behavioral measure of alcohol consumption was added following cue exposure. Moreover, the risk groups in Study 1 were defined in terms of quantity and frequency of consumption, rather than broader drinking criteria which also capture the consequences of alcohol use. To address this limitation, an alternative method to define alcohol-related risk status was used in Study 2. The Alcohol Use Disorders Identification Test (AUDIT; Saunders et al., 1993) takes broader characteristics of risky alcohol use into account, such as the extent to which it leads to harmful behavior or possible dependence. Another limitation in Study 1 was that there was no control group where participants were exposed to a similar craving induction procedure but with non-alcoholic beverage cues. Therefore, it is unclear if the craving profiles with respect to relative approach and avoidance inclinations in the present study reflect a reactivity specific to alcohol cues. In Study 2, a comparison condition was included which used the same exposure procedure but instead using non-alcoholic cues.

## Study 2

Alcohol consumption following cue exposure in the lab typically involves implementing a taste test (e.g., Weafer and Fillmore, 2008; Houben et al., 2010; Jones et al., 2011; Fernie et al., 2012; Schlauch et al., 2015a). As yet, there is limited evidence whether cue-elicited changes in approach and avoidance inclinations are associated with subsequent alcohol consumption. In one recent study, Jones et al. (2013) measured approach and avoidance with the multi-item Approach and Avoidance of Alcohol Questionnaire (McEvoy et al., 2004) before and after alcohol exposure and administered a subsequent taste test in heavy drinkers. However, avoidance ratings had been extremely low prior to cue exposure and remained unchanged throughout the subsequent tasks prior to the taste test, and were not associated with alcohol consumed in the taste test. Approach inclinations had increased in the alcohol exposure condition, but decreased again to pre-exposure levels by the time the taste test was administered, and were positively associated with alcohol consumption in both the alcohol cues group and the water cues control group. This makes interpretation difficult and it remains unclear if relative changes in craving profiles are associated with subsequent alcohol consumption and if this is specific to alcohol cue exposure.

Hence, the first aim of Study 2 was to include a taste test to measure the influence of approach and avoidance, and the relative balance of these inclinations, on alcohol consumption. The second aim was to replicate the pattern of approach and avoidance inclinations found in Study 1, but to select high and low risk drinkers based on cut-off scores on a broader risk screening measure (AUDIT; Saunders et al., 1993) rather than the recommended drinking limits based on only frequency and quantity of alcohol consumption used in Study 1. The final aim was to include a non-alcohol control condition to determine whether the pattern of inclination change observed in Study 1 was unique to the alcohol condition.

Predictions regarding the three possible patterns of changes in the relative strength of approach on avoidance inclinations for alcohol are identical to Study 1, except these changes are specific to the alcohol cue exposure condition. That is, there would be an interaction with exposure condition such that, compared to low risk drinkers, high risk drinkers would shift to greater alcohol approach inclinations relative to alcohol avoidance inclinations only in the alcohol exposure condition but not in the non-alcohol exposure condition. With respect to the taste test, high risk drinkers when given the opportunity would be expected to drink more alcohol than low risk drinkers across both exposure conditions, but there would also be a risk group by exposure condition interaction because the influence of alcohol cue exposure on alcohol consumption should be stronger in high risk drinkers than in low risk drinkers. Finally, the critical feature that distinguished low from high risk drinkers in their cue-elicited craving profiles in Study 1 was that avoidance inclinations strongly counterbalanced increased approach inclinations only in the low risk drinkers, whereas approach was dominant over avoidance in the high risk drinkers. If the post-exposure synergy of elevated approach and diminished avoidance characterizes high risk drinkers (cf. Schlauch et al., 2013), then cue-elicited changes in both approach and avoidance inclination should predict alcohol consumption at the subsequent taste test in that group. In contrast, if the antagonistic role of equally strong avoidance balancing increased approach characterizes low risk drinkers, neither approach nor avoidance inclinations on their own may predict subsequent alcohol consumption.

### Materials and Methods

#### Participants

Participants were 79 (69% female) undergraduate students aged between 18 and 34 (*M* = 19.15, *SD* = 2.21) from UWA and assigned to the low risk group if they scored between one and seven on the AUDIT (indicating “low risk” drinking), and to the high risk group if they scored 12 and above on the AUDIT (indicating “risky” to “very risky” drinking) (Babor et al., 2001). Those in the low risk AUDIT group reported an average weekly alcohol intake of 2.5 (*SD* = 4.2) standard drinks, compared to 33.83 (*SD* = 66.71) standard drinks for the high risk AUDIT group. To simplify the materials and procedures associated with the craving manipulation and taste test, only participants who reported at least some beer consumption were eligible. Participants in both low and high risk groups were randomly allocated to either an alcohol or non-alcohol (control) craving induction condition. This resulted in 20 high risk (45% female) and 20 low risk (60% female) participants in the alcohol condition, and 19 high risk (32% female) and 20 low risk (65% female) participants in the non-alcohol condition.

#### Materials

As per Study 1, the DBQ and craving items (approach and avoidance) were included in Study 2. An additional question on the demographic questionnaire ascertained a non-alcoholic beverage preference (soft drink or juice).

##### Alcohol Use Disorders Identification Test (AUDIT; Saunders et al., 1993)

The AUDIT includes 10 items which were used to categorize low and high risk drinkers. Scores on the AUDIT range from 0 to 40. Scores of 1–7 indicate low risk drinkers, and scores of 8+ indicate risky drinkers. Scores above 15 are classified as very risky (Babor et al., 2001). The cut-off score for the high risk group in the current study was 12, representing at least the high range of the risky drinking category. The AUDIT captures risky drinking across three domains: hazardous use, dependence symptoms, and harmful use. Hazardous use refers to a drinking pattern associated with a risk of harmful consequences; dependence symptoms refers to the cognitive, behavioral, and physiological symptoms due to alcohol use; and harmful use refers to a drinking pattern which causes physical and psychological damage to an individual’s health (Bohn et al., 1995). As a unidimensional estimate of risky drinking, the AUDIT offers excellent internal consistency (α = 0.94 in our screening sample; *N* = 824).

##### The alcohol craving induction

The alcohol craving induction materials remained the same as in Study 1, except that to simplify the materials required for the following taste test, only beer imagery was used in the alcohol condition. A non-alcohol condition was also included whereby two additional still and video montages were available which matched the beer imagery but presented soft drink or juice depending on the participant’s preference. To match the alcohol condition, there were soft-drink or juice cartons stacked next to the fridge and an A3-sized poster of non-alcoholic beverages.

##### The taste test

A taste test was administered as a behavioral measure of alcohol consumption following the craving induction. The experimenter poured 330 mL of beer and 330 mL of the participants’ preferred non-alcoholic beverage (orange juice, apple juice, Coca-Cola, or lemonade) into two tall glasses. These glasses were marked at the 330 mL level to ensure accuracy, although this mark faced away from participants. The order of pouring the beer and non-alcoholic beverages was counterbalanced across participants. “Rating sheets” for each beverage were given to participants for them to rate aspects such as flavor and carbonation. Participants were also asked to estimate the percentage of alcohol of the beer they had tasted. Specifically, they were instructed, “Australian beers come in three standard strength categories (low, mid and full) that represent 2.7, 3.5, and 4.8% alcohol content respectively. Some beers can have higher or lower alcohol content than is typical of Australian beers. In this question please estimate the percentage of alcohol in the beer you have tasted.” The response options were: *Less than* 2.7, 2.7, 3.5, 4.8%, *more than* 4.8%. The alcohol content estimate was analyzed to assess whether participants were aware that a non-alcoholic beer had been used for the taste test. Analysis revealed that 85.9% of the sample selected 2.7% or greater. The brand of beer used was Clausthaler, with an alcohol percentage of 0.45%, classifying it as non-alcoholic (U.S. Customs and Border Protection, 2008). Using a non-alcoholic beer ensured that participants were not at risk of being intoxicated after the experiment. The beer was chosen based on pilot testing, where ten participants blindly compared three non-alcoholic beers, namely Clausthaler (0.45%), Holsten (0.02%), and Coopers (0.7%). Of the three beer types, both Clausthaler and Coopers were estimated to have a higher alcohol content (more than 3% alcohol) than Holsten (less than 3% alcohol), but Clausthaler was more popular than Coopers, as determined by the average volume consumed (90.62 mL vs. 49.12 ml). For the taste test, all labels were removed and caps blacked out so the brand was unidentifiable. Using the same procedures as in Fernie et al. (2012), participants were instructed to drink “as much or as little as they like” to make the ratings and were not given a time limit. However, all participants completed the task within 10 min. Beer consumption was measured in two ways, as a percentage of the total fluid consumed, which provides a relative measure of alcohol consumption (combined with non-alcoholic consumption), and as the volume of alcohol consumed (mL), which provides an absolute measure of alcohol consumption (cf. Jones et al., 2016). A moderate correlation of *r* = 0.52 (*p* < 0.001) was found between these two outcome measures, suggesting that the two only share 27% of variance, and therefore offer distinct representations of alcohol consumption behavior.

#### Procedure

The same recruitment procedure was used as in Study 1, except that in Study 2 the administration of the AUDIT accompanied the DBQ during the pre-screen session. The laboratory procedures were identical to those in Study 1 except that in Study 2, following the second craving measurement (Time 2), participants were given the taste test after which they completed a third craving measurement (Time 3). These procedures were approved by the UWA Human Research Ethics Committee.

### Results

Descriptive statistics for AUDIT scores and self-reported alcohol use for high and low risk groups in each exposure condition are summarized in **Table 1**.

**Table 1:** Summary statistics for AUDIT scores and self-reported alcohol use for each group based on risk status and craving condition.

	High Risk				Low Risk				Main effect of AUDIT group	
	Alcohol		Non-alcohol		Alcohol		Non-alcohol			
**Variable**	***M***	***SD***	***M***	***SD***	***M***	***SD***	***M***	***SD***	***F* (1,75)**	**$\eta_{p}^{2}$**

AUDIT total	16.6^a^	4.18	16.58^a^	5.07	4.25^b^	1.94	4.65^b^	1.69	236.71^∗∗∗^	0.76
Drinks/Week	39.20^a^	68.11	28.17^a^	66.57	2.95^b^	2.53	2.08^b^	2.73	8.34^∗∗^	0.10

*Same superscripts denote no significant difference, different superscripts denote significant difference. ^∗∗∗^*p* < 0.001, ^∗∗^*p* < 0.01.*

#### Craving Manipulation and Change in Approach and Avoidance Inclinations

**FIGURE 3:**
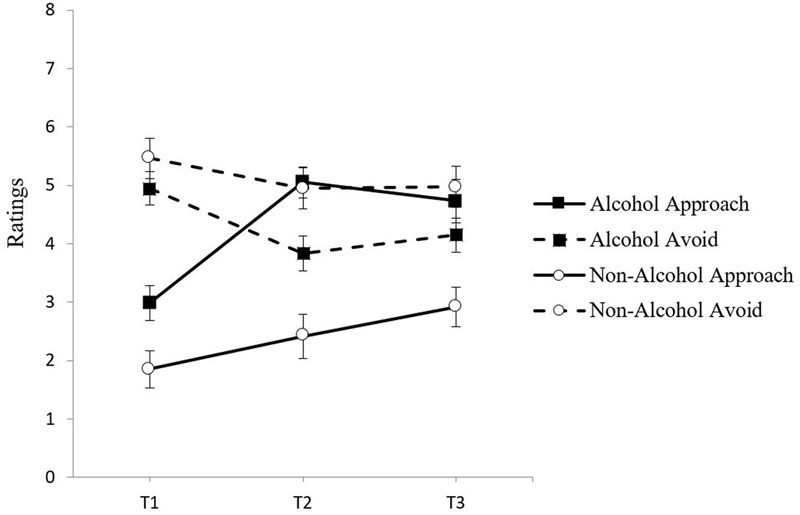
**Mean approach and avoidance inclinations at Time 1, Time 2, and Time 3 in the alcohol and non-alcohol exposure conditions with standard error bars**.

To evaluate the pattern of change in approach and avoidance inclinations following the craving manipulation through to the subsequent taste test, a 2 × 2 × 3 × 2 mixed-model ANOVA (Group × Condition × Time × Inclination) was conducted with group (low and high risk) and condition (alcohol and non-alcohol) as between-groups factors, and time (pre cue exposure, post-cue exposure, and post-taste test) and inclination (approach and avoidance) as within-groups factors. The assumption of sphericity, examined via Mauchley’s test was rejected; therefore, Greenhouse–Geisser corrected *F*-tests have been reported. The four-way interaction was not significant, *F*(1.47,110.23) = 2.87, *p* = 0.077, $\eta_{p}^{2}$ = 0.04. There were significant main effects of condition, *F*(1,75) = 5.67, *p* < 0.05, $\eta_{p}^{2}$ = 0.07, time, *F*(1.80,134.84) = 8.95, *p* < 0.001, $\eta_{p}^{2}$ = 0.11, and inclination, *F*(1,75) = 18.22, *p* < 0.001, $\eta_{p}^{2}$ = 0.20, which were qualified by a significant three-way interaction between condition, time, and inclination, *F*(1.47,110.23) = 4.98, *p* < 0.05, $\eta_{p}^{2}$ = 0.06. **Figure 3** illustrates that, across both risk groups, participants showed a pattern of increased approach and decreased avoidance from Time 1 to Time 2, with this effect being more pronounced in the alcohol condition. Paired samples *t*-tests showed that for participants in the alcohol condition, there was a significant increase in approach from Time 1 to Time 2, *t*(39) = -11.98, *p* < 0.001, *d* = -1.16, which was maintained at Time 3 compared to Time 1, *t*(39) = -5.13, *p* < 0.001, *d* = -0.82). Furthermore, these individuals showed a significant decrease in avoidance from Time 1 to Time 2, *t*(39) = 5.15, *p* < 0.001, *d* = 0.60, which was maintained at Time 3, *t*(39) = 3.71, *p* < 0.01, *d* = 0.44. For the non-alcohol condition, while there were also increases in approach, *t*(38) = -3.86, *p* < 0.001, *d* = -0.26, and decreases in avoidance, *t*(38) = 2.80, *p* < 0.01, *d* = 0.23, from Time 1 to Time 2, examination of the effect sizes show that the magnitude of the effect in the alcohol condition was four times larger for approach (*d_alcoholT*1*-T*2*_* = -1.16 vs. *d_non-alcoholT*1*-T*2*_* = -0.26) and over two times larger for avoidance (*d_alcoholT*1*-T*2*_* = 0.60 vs. *d_non-alcoholT*1*-T*2*_* = 0.23). This demonstrates that the craving induction procedure was effective, resulting in a greater magnitude of change in approach and avoidance in the alcohol condition compared to the non-alcohol condition. It also shows that these changes in craving dimensions were maintained until the time of the taste test. The alcohol condition was examined next.

**FIGURE 4:**
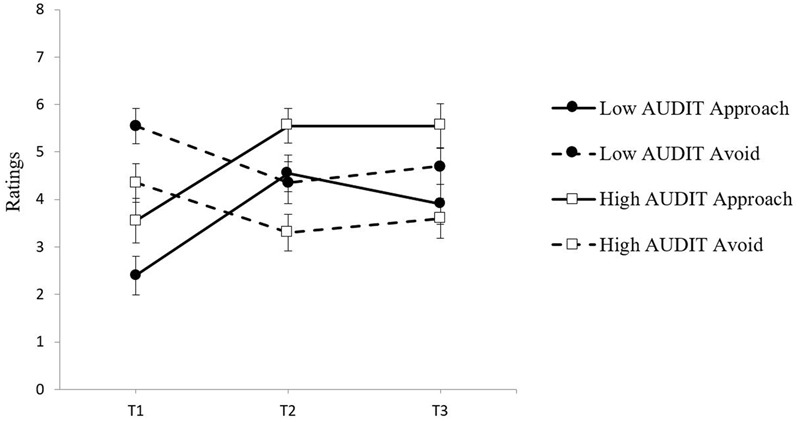
**Approach and avoidance inclination means at Time 1, Time 2, and Time 3 for both groups in the alcohol exposure condition with standard error bars**.

To evaluate the pattern of change in approach and avoidance inclinations from pre- to post-craving induction in the alcohol condition, a 2 (Group: High and Low Risk) × 2 (Time: 1 and 2) × 2 (Inclination: Approach and Avoidance) mixed model ANOVA was conducted with group as the between-groups factor, and time and craving inclination as the within-groups factors.. There was a significant Time × Inclination interaction, *F*(1,38) = 87.69, *p* < 0.001, $\eta_{p}^{2}$ = 0.70, indicating that the pattern of change in approach and avoidance inclinations differed following the craving induction. **Figure 4** illustrates that both risk groups showed a pattern of increased approach and decreased avoidance from Time 1 to Time 2. The decrease in avoidance was similar for the high risk group *t*(19) = 3.28, *p* < 0.01, *d* = 0.59 and the low risk group, *t*(19) = 3.94, *p* < 0.01, *d* = 0.66. The increase in approach was also similar for the high risk group, *t*(19) = -8.31, *p* < 0.001, *d* = -1.07, and the low risk group *t*(19) = -8.46, *p* < 0.001, *d* = -1.39. This pattern is similar to Study 1 findings although the low risk group in Study 1 did show a somewhat smaller decrease in avoidance. This is consistent with aspects of pattern (1) because both high and low risk drinkers displayed a pattern of increased approach and a significant decrease in avoidance following alcohol cue exposure. However, as in Study 1, the low and high risk groups also differed at baseline. As can be seen in **Figure 4**, both groups had higher avoidance than approach ratings at Time 1, but this difference was again much larger for the low risk group. This is consistent with pattern (3) requiring a closer inspection of how changes in the relative balance of approach and avoidance from Time 1 to Time 2 may yield distinct craving profiles for each risk group post-craving induction.

#### Relative Balance Assessment of Approach and Avoidance

To examine how the relative strength of approach and avoidance inclinations changed from baseline to post-cue exposure, inclinations were examined separately for Time 1 and Time 2, using 2 × 2 (Group × Inclination) ANOVAs. At Time 1, there was a main effect of inclination, *F*(1,38) = 18.54, *p* < 0.001, $\eta_{p}^{2}$ = 0.33, and there was also a Group × Inclination interaction, *F*(1,38) = 6.56, *p* < 0.05, $\eta_{p}^{2}$ = 0.15. Paired-sample *t*-tests revealed that the low risk group showed greater avoidance than approach at Time 1, *t*(19) = -5.22, *p* < 0.001, *d* = -1.99, whereas for the high risk group this difference between inclinations was nearly five times smaller and non-significant, *t*(19) = -1.16, *p* = 0.261, *d* = -0.41. At Time 2, replicating the pattern found in Study 1, the relative strength of approach and avoidance inclinations had again *reversed* for the two groups as evidenced by a significant Group × Inclination interaction, *F*(1,38) = 5.07, *p* < 0.05, $\eta_{p}^{2}$ = 0.12. Paired samples *t*-tests confirmed that whereas for the high risk group approach was now much stronger than avoidance at Time 2, *t*(19) = 3.76, *p* < 0.01, *d* = 1.34, for the low risk group, approach was still balanced by equally strong avoidance at Time 2, *t*(19) = 0.29, *p* = 0.774, *d* = 0.11.

**FIGURE 5:**
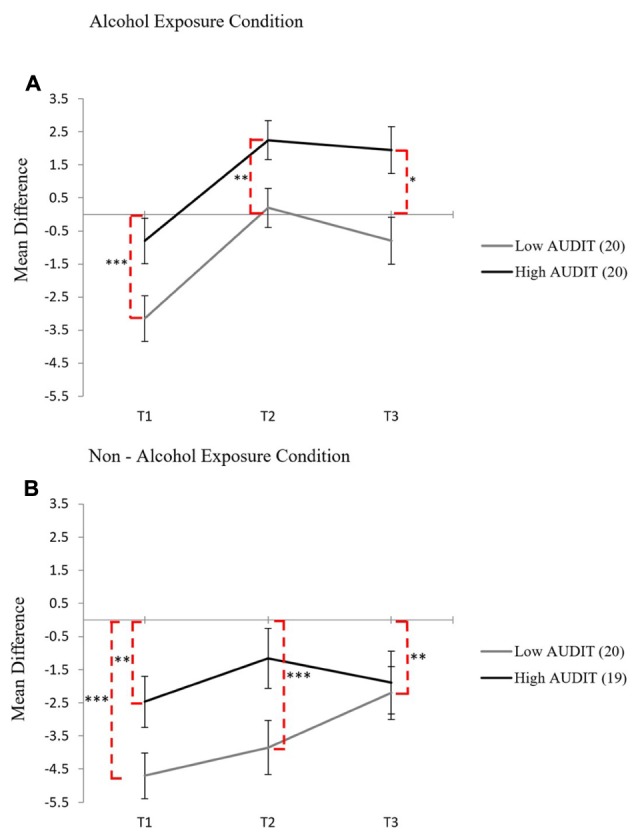
**Mean difference scores for each group in the alcohol cue exposure condition (A)** and in the non-alcohol exposure condition **(B)** across Time 1, Time 2, and Time 3 with standard error bars, dashed lines indicate significance from zero (^∗^*p* < 0.05, ^∗∗^*p* < 0.01, ^∗∗∗^*p* < 0.001).

This reversal in the relative balance of approach and avoidance inclinations following alcohol cue exposure was again further examined using approach-avoidance difference scores across each risk group. Three time points were calculated, including a measurement following the taste test (Time 3). **Figure 5A** illustrates that in the alcohol condition, at Time 1, for low risk drinkers the negative difference score was significantly different from zero, *t*(19) = -5.22, *p* < 0.001, *d* = -1.15, showing that the balance between approach and avoidance is tipped in favor of avoidance, whereas for high risk drinkers the negative difference score was not significantly different from zero, *t*(19) = -1.16, *p* = 0.261, *d* = -0.26, showing that there was an equal balance between approach and avoidance. At Time 2, while the net strength of approach had increased for both groups, only for the high risk drinkers was there now a positive difference score that was significantly different from zero, *t*(19) = 3.76, *p* < 0.01, *d* = 0.84, showing that the initial balance at Time 1 between approach and avoidance had tipped in favor of approach. In contrast to high risk drinkers, while the low risk drinkers have also shifted to a positive difference score, it was not significantly different from zero, *t*(19) = 0.29, *p* = 0.774, *d* = 0.07. That is, while the net strength of approach had also increased, it was still balanced by an equally strong avoidance inclination. **Figure 5B** shows that in the non-alcohol control condition difference scores were negative for both high risk and low risk drinkers throughout the craving induction, showing that alcohol avoidance inclinations remained predominant over alcohol approach inclinations for both risk groups when no alcohol cue exposure was present.

#### Alcohol Consumption Volume (ml)

To test the prediction that a greater alcohol volume (mL) was consumed by high risk drinkers and that there would be an interaction between group and condition, a 2 × 2 (Group × Condition) between groups ANOVA was conducted. Analysis of the volume of beer consumed showed that high risk drinkers drank more beer, (*M* = 107.25 mL, *SD* = 79.34), than low risk drinkers, (*M* = 70.68 mL, *SD* = 62.14), across both conditions, *F*(1,75) = 5.13, *p* < 0.05, $\eta_{p}^{2}$ = 0.06. There was no main effect of condition, *F*(1,75) = 0.77, *p* = 0.38, $\eta_{p}^{2}$ = 0.01), and no Group × Condition interaction, *F*(1,75) = 0.25, *p* = 0.617, $\eta_{p}^{2}$ = 0.00, for the volume (mL) of alcohol consumed.

As there was a main effect of risk group on beer volume (mL), two separate hierarchical regressions were conducted to examine whether approach and avoidance predicted beer consumption for high and low risk drinkers. While the effect of condition on beer volume (mL) was non-significant, condition was entered at Step 1 to control for any potential residual variance due to condition. For the high risk group, where approach inclinations were higher than avoidance inclinations following cue exposure, Time 2 inclinations significantly predicted beer consumption, *R* = 0.59, *R*^2^= 0.35, *R_Δ_^2^* = 0.35, *p* < 0.001, *F*(3,35) = 6.26, *p* < 0.01. Examination of standardized beta coefficients revealed that approach inclinations (β = 0.39, *p* < 0.001), and avoidance inclinations (β = -0.36, *p* < 0.05) were significant predictors. The *R* (0.04) and *R*^2^ (<0.01) at Step 1 were non-significant (*p* = 0.816). In contrast, for the low risk group, where increased approach was still balanced by avoidance, Time 2 inclinations did not significantly predict beer consumption, *R* = 0.29, *R*^2^= 0.08, *R_Δ_^2^* = 0.05, *p* = 0.394, *F*(3,36) = 1.07, *p* = 0.376. The *R* (0.18) and *R*^2^ (0.03) at Step 1 were non-significant (*p* = 0.263). In sum, both inclinations significantly predicted consumption in high risk drinkers, a synergistic effect, whereas neither did so in low risk drinkers, a canceling out – or antagonistic effect.

It is also noteworthy that while both risk groups showed a comparable increase in approach inclinations following the cue exposure at Time 2, only low risk drinkers showed a subsequent decrease in approach inclinations by Time 3 after they had consumed alcohol during the taste test, whereas high risk drinkers showed no decline in approach inclinations after alcohol consumption (see **Figure 4**). Linear trend analyses from Time 1 to Time 3 confirmed that for the low risk group the quadratic effect, *F*(1,19) = 30.03, *p* = 0.000, $\eta_{p}^{2}$ = 0.61, was nearly twice as strong as the linear effect, *F*(1,19) = 9.40, *p* = 0.006, $\eta_{p}^{2}$ = 0.33. For the high risk group, the quadratic effect, *F*(1,19) = 17.27, *p* = 0.001, $\eta_{p}^{2}$ = 0.48, was identical to the linear effect, *F*(1,19) = 17.27, *p* = 0.001, $\eta_{p}^{2}$ = 0.48, and smaller than for the low risk group ($\eta_{p}^{2}$ = 0.476 versus 0.613). This change in direction of approach inclinations for low risk drinkers after the taste test suggests that the opportunity to consume a small amount of alcohol may have been sufficient to partly satiate the cue-elicited approach inclinations in the low risk drinkers, but not in the high risk drinkers.

#### Proportion of Alcohol Consumed Relative to Total Fluid

To test the prediction that a greater proportion of alcohol (relative to total fluid consumed) was consumed in the alcohol condition, and that there would be an interaction between group and condition, a 2 × 2 (Group × Condition) between groups ANOVA was conducted. Analysis of the proportion of beer consumed relative to total fluid consumed showed that those in the alcohol condition drank proportionally more beer, (*M* = 53.3%, *SD* = 24.3), than those in the non-alcohol condition, (*M* = 43.7%, *SD* = 20), across both groups, *F*(1,75) = 3.54, *p* = 0.06, $\eta_{p}^{2}$ = 0.05, although this effect was just outside conventional margins of significance. There was no main effect of group, *F*(1,75) = 0.14, *p* = 0.712, $\eta_{p}^{2}$ = 0.71, and no Group × Condition interaction, *F*(1,75) = 0.003, *p* = 0.954, $\eta_{p}^{2}$ = 0.00, for the proportion of alcohol consumed.

### Discussion

The pattern of change in approach-avoidance craving profiles following alcohol cue exposure is consistent with the pattern observed in Study 1. That is, in line with pattern (3), despite similar increases in approach, high and low risk groups had different craving profiles following alcohol exposure because of their distinctly different relative balance profiles prior to alcohol exposure. Specifically, high risk drinkers were characterized by considerably less elevated avoidance relative to approach inclinations compared to low risk drinkers at baseline. As such, the change in the strength in approach overtook the strength of avoidance in high risk drinkers, but was not sufficient to overcome the strength of avoidance in low risk drinkers. These important differences only emerge when examining simultaneous changes in the relative strength of approach and avoidance dimensions of craving. Moreover, the synergistic effect of increased approach and decreased avoidance inclinations in high risk drinkers predicted alcohol consumption, but neither inclination predicted consumption in low risk drinkers where approach inclinations were countered by equally strong avoidance inclinations.

## General Discussion

The ambivalence model of craving (Breiner et al., 1999), posits that craving involves the dynamic interplay of separate approach and avoidance inclinations. These two inclinations may act synergistically resulting in determined craving states if one inclination dominates over the other; or they may act antagonistically resulting in ambivalent craving states. Predominant avoidance or ambivalent craving profiles are more likely to result in restraint, whereas predominant approach is more likely to result in alcohol consumption and potentially risky drinking if unimpeded by restraint.

In two experiments designed to track cue-elicited changes in alcohol approach and avoidance inclinations over time we found distinct craving profiles for low and high risk drinkers. While both groups showed comparable increases in approach inclinations after alcohol cue exposure, the relative strength of competing avoidance inclinations differed across risk groups. Specifically, prior to cue exposure, both groups reported higher avoidance than approach inclinations, but this difference was much smaller for the high risk drinkers. This is important because these different starting points have a bearing on the relative balance following cue exposure, even if the magnitude of change in approach and avoidance inclinations is equivalent for both risk groups. In both studies, analyses of approach-avoidance difference scores showed that cue-elicited craving was characterized by a predominant approach inclination only in the high risk drinkers, whereas in the low risk drinkers the cue-elicited increase in approach inclinations was balanced by an equally strong avoidance inclinations. In the latter antagonistic post-cue exposure profile for the low risk drinkers, neither inclination predicted subsequent amount of alcohol consumed during the taste test, whereas each inclination predicted subsequent drinking in the high risk drinkers. Moreover, even though low risk drinkers consumed less alcohol during the taste test than high risk drinkers, alcohol consumption during the taste test resulted in a subsequent decline in approach inclinations in the low risk drinkers, but not in the high risk drinkers. In sum, assessment of the relative balance of approach and avoidance inclinations to use alcohol following cue exposure reveals changes in craving profiles that are not apparent from examining changes in approach inclinations alone, and it is this shift in the net balance that distinguishes high from low risk drinkers.

The pattern of increasing predominance of approach over avoidance inclinations following cue exposure evident in the high risk drinkers was recently also found consistently across three samples of heavy drinkers following a positive-alcohol video priming procedure, which highlighted positive consequences and associations with alcohol drinking (Di Lemma et al., 2015). Unlike the risky drinkers in the current studies, heavy drinkers in all three experiments already had higher approach than avoidance inclinations prior to the cue exposure, and that gap in the net balance became even wider following exposure to the positive-alcohol video. In contrast, heavy drinkers assigned to a negative-alcohol video priming condition, which highlighted negative consequences of drinking including graphic depictions used in government warning messages, showed a large reverse shift in the net balance of approach and avoidance inclinations following the video. Their craving profile shifted from dominant approach to a pattern where decreases in approach and simultaneous increases in avoidance resulted in a balanced ambivalent craving profile, similar to that shown by low risk drinkers in the present studies. This suggests that for heavy drinkers a negative priming procedure may be required to change the net craving profile such that approach inclinations are held in check by equally strong avoidance inclinations. By contrast, for the low risk drinkers in the present studies no such intervention is necessary to maintain a balanced net craving profile despite increases in approach inclinations following an alcohol cue exposure which did not highlight either positive or negative consequences. Thus, low risk drinkers are more likely to react to alcohol cues with a tempered or ambivalent craving profile, whereas high risk drinkers respond to the same cues with a determined, approach-oriented craving profile.

There were two notable limitations in Study 2. Firstly, because of the use of a non-clinical sample, the current findings may not generalize to individuals with more severe alcohol use problems or those who experience dominant resting approach inclinations. For example, while participants with the highest AUDIT scores were prioritized during recruitment, the average score for the high risk drinkers in Study 2 was 16.59 (*SD* = 4.57), which represents the low range of very risky drinkers. Furthermore, in both Studies 1 and 2, the high risk drinkers showed dominant avoidance inclinations at baseline, which contrasts to the dominant baseline approach inclinations seen in other risky drinking samples, such as those used by Di Lemma et al. (2015). These findings suggest that baseline craving might be highly variable across non-clinical samples. A second limitation was the inconsistency in outcomes across the two alcohol consumption measures. Beer consumed as a proportion of the total fluid offered, a relative measure, yielded an effect of condition but not risk group. By contrast, alcohol volume, an absolute measure, yielded an effect of risk group but not condition. Alcohol consumed as a proportion of total fluid is the more commonly used index of consumption following a taste test and, similar to the present study, has yielded consistent condition effects following some form of alcohol exposure or avoidance/restraint training (Field and Eastwood, 2005; Christiansen et al., 2013; Jones and Field, 2013; Jones et al., 2013). However, these studies did not compare different risk groups, and the failure of the relative consumption measure to distinguish between the high and low risk groups in the present study may suggest that it is less sensitive to detect differences based on risk status. In contrast, the absolute consumption measure based on the alcohol volume consumed differentiated between our high and low risk groups. Fernie et al. (2012) also used absolute volume of alcohol consumed to compare different risk groups (heavy versus moderate drinkers) following an alcohol priming procedure, but did not find a difference between groups. However, the low risk group in our Study 2 was characterized by a considerably lower weekly mean standard drink intake than the moderate drinkers in the study by Fernie et al. (2012, 2.5 versus 13.27), even if accounting for differences in standard drink criteria in Australia (10 g of alcohol) versus the United Kingdom (8 g of alcohol) (Miller et al., 1991). It is possible that differences in alcohol volume consumed following a taste test between risks groups only emerge when comparing very low risk individuals to those at more extreme levels of risk. Indeed, the high risk drinkers in our studies were characterized by similar weekly mean standard drink intake to the high risk drinkers in the study by Fernie et al. (2012, 33.17 versus 31.74), suggesting that increased disparity in risk status is necessary to detect these *ad libitum* alcohol volume consumption effects. It is therefore recommended that future studies explore both outcome alcohol consumption measures in groups with differing risk status to establish whether these measures offer varying utility for detecting experimental versus risk status effects.

Notwithstanding these limitations, the current findings provide further validation for the utility of an approach-avoidance conceptualization of craving. Across two experiments results confirmed that assessment of the relative balance of approach and avoidance inclinations to use alcohol following cue exposure reveals changes in craving profiles that are not apparent from examining changes in approach inclinations alone, and it is this shift in the net balance which distinguishes high from low risk drinkers. The synergistic or antagonistic fluctuations in the relative strength of approach and avoidance inclinations are a promising avenue for future research on how individual differences in cue-elicited craving impact on harmful drinking and recovery from alcohol use disorders.

## Author Contributions

RH, WS, PE, and MW all made substantial contributions to the conception of the research and interpretation of the data. RH and WS were heavily involved in preparing the drafts of the manuscript while PE and MW provided comments on the final revisions. All four authors reviewed and approved of the final manuscript to be submitted. All four authors agree to be accountable for all aspects of the work to ensure that questions related to the accuracy or integrity of any part of the work are appropriately investigated and resolved.

## Conflict of Interest Statement

The authors declare that the research was conducted in the absence of any commercial or financial relationships that could be construed as a potential conflict of interest.
